# Exploring the meta-motivational strategies utilized by medical students in Jordan: an exploratory study

**DOI:** 10.1186/s12909-025-08189-1

**Published:** 2025-11-18

**Authors:** Rand Murshidi, Mahmoud Abdallat, Muhammad Hammouri, Rand Al-Huneidy, Khaled Alenezi, Abdulhadi Alrajehi, Nawal Al-Mutairi, Waleed Alkanderi, Abdulwahab Alkandari, Abdulrahman Aldousari, Sara Alenezi, Ahmad Taleb, Sayed Alzalzaleh, Adnan Alkayal, Hana Taha, Abdallah Al-Ani

**Affiliations:** 1 Department of Dermatology, The University of Jordan, Amman, Jordan; 2 Department of Neurosurgery, The University of Jordan, Amman, Jordan; 3 Department of Neurology and Neurosurgery Montreal Neurological Institute, McGill University, Montreal, Canada; 4 School of Medicine, The University of Jordan, Amman, Jordan; 5 Al-Zarqaa’ Governmental Hospital, Al Zarqaa’, Jordan; 6 Department of Family and Community Medicine, School of Medicine, The University of Jordan, Amman, Jordan; 7 Department of Neurobiology, Care Science and Society, Karolinska Institutet, Stockholm, Sweden; 8 Office of Scientific Affairs and Research, King Hussein Cancer Center, Amman, Jordan

**Keywords:** Meta-motivation, Medical education, Medical students, Self-regulation

## Abstract

**Background:**

Meta-motivational strategies refer to the ability to monitor and adapt one’s motivational state to accomplish certain goals and are highly significant in medical students due to their unique educational environment. The utilization of such strategies has not been previously studied in Jordanian medical students.

**Methods:**

A cross-sectional design surveyed 409 students using the Meta-Motivational Strategies in Medical Students Questionnaire (MSMQ), assessing seven domains: regulation of value, environmental structuring, relatedness, promotional/preventional situational awareness, situational interest, and self-consequencing.

**Results:**

Key findings revealed regulation of value and environmental structuring as dominant strategies, aligning with societal emphasis on education and adaptive responses to academic rigor. Male students scored significantly higher in regulation of relatedness (MD: -0.79, *p* < 0.05). Students living alone demonstrated stronger environmental structuring (MD: 1.16, *p* < 0.05) and self-consequencing (MD: 0.68, *p* = 0.024). Students who chose to enroll in medicine autonomously scored higher across most strategies (*p* < 0.05). Students who participated in research activities exhibited greater regulation of value and situational interest (*p* < 0.05). GPA disparities highlighted that high achievers (Excellent GPA) scored higher in regulation of value and environmental structuring (*p* < 0.05). No differences emerged between first-generation and non-first-generation students.

**Conclusion:**

These findings emphasize cultural, institutional, and psychological influences on meta-motivation; by bridging these gaps, educators can develop targeted interventions to foster adaptive meta-motivational strategies, ultimately supporting student well-being and success in medicine.

## Introduction

Successful students employ targeted strategies to sustain their motivation, as it is often described as one of the cornerstones of learning [1, 2]. Medical education is globally recognized as exceptionally demanding, as it calls for constant motivation, flexibility, and psychological fortitude. Academic success, personal well-being, and long-term professional development can all be significantly impacted by students’ ability to regulate their motivation in such a demanding setting [3]. Regulating motivation involves the conscious effort to manage or alter motivation to accomplish a specific challenging task [4]. Strategies pertaining to self-regulating motivation have a positive influence on fostering perseverance and improving future performance [5]. According to the Self-Determination Theory (SDT), cultivating autonomous motivation in medical students is key to building life-long learners–a critical trait in an ever-evolving field [6]. Autonomous motivation is characterized as the most optimal form of motivation, where students engage in learning in response to genuine interest or personal significance [7]. In medical education, where students are facing intense academic pressures, clinical uncertainties, and emotional fatigue, the ability to self-regulate motivation is indispensable for long-term success and well-being.

Studies discussing motivation mainly emphasize intrinsic versus extrinsic factors [8, 9]. In a 30-month longitudinal study, Da Silva et al. demonstrated a consistent decline of intrinsic and extrinsic motivation across 303 medical students and concluded that motivation deteriorates across different years of medical school [10, 11]. Similarly, Del-Ben et al. reported a significant decrease in the intrinsic motivation of first-year medical students [12]. These studies indicate that fostering a meaningful impact on motivation in medical students necessitates addressing not only external and environmental factors but also internal determinants, as the literature posits that these elements are deeply intertwined [13].

Meta-motivation, which is the ability to monitor and adapt one’s motivational state to accomplish certain goals, emerges as an important concept particularly relevant to medical education settings [14]. As medical education often presents challenges that are unique in their nature due to transitions from different phases from theoretical science to clinical practice, and with each phase having specific demands, recent literature indicates the role of many meta-motivational strategies in sustaining and improving motivation in this field [15].

Despite growing literature on the subject, there remains a paucity of evidence regarding the active role of students in managing and controlling their motivation, particularly when highlighting different educational and cultural contexts. Within a Jordanian context, limited evidence exists pertaining to the motivational thresholds of healthcare students. Khamaiseh et al. demonstrated that academic motivation is generally below international averages among nursing students [16]. Moreover, it is unknown which components of motivation are targeted by medical students when utilizing any meta-motivational strategy [17]. Thus, concerned policy makers are unable to cater to the cognitive needs of prospective students due to the lack of evidence on the status of motivation or its links to students’ academic desires or intentions.

Therefore, this study aims to explore meta-motivational strategies employed by medical students primarily in Jordan and how various demographic factors may influence the use of said strategies. Furthermore, it seeks to identify potential predictors of strategy utilization across numerous social and demographic dimensions.

## Methodology

### Design and participants

We conducted a cross-sectional investigation of meta-motivational strategies employed by medical students enrolled at six Jordanian universities. Data were collected from November to December 2024. We utilized an online, self-administered questionnaire formulated on Google Forms. The questionnaire was disseminated using social media (e.g., Facebook and WhatsApp) to official groups of targeted medical students at the University of Jordan, Jordan University of Science and Technology, the Hashemite University, Mutah University, Yarmouk University, and Zarqa University. We employed convenience and snowball sampling to reach the required sample size.

### Instrument

The Meta-motivational Strategies in Medical Students questionnaire (MSMQ) was utilized in our study. Developed by Norouzi et al., the questionnaire is comprised of 28 items across 7 domains. Items were assessed on a 5-point Likert scale ranging from ‘never’ to ‘always’. The content validity of the questionnaire had an index of 0.79. The reliability, represented by Cronbach alpha coefficient, was measured at 0.89 with an intra-class correlation coefficient of 0.87 [18]. The questionnaire was disseminated using its original English version.

The questionnaire was categorized to the following domains: the regulation of value domain examined how students perceive the significance of medical education and its relation to their future careers, as well as how they prioritize time for important subjects to manage their academic motivation. The environmental structuring domain was concerned with fostering a focused and a supportive study environment. The regulation of relatedness domain highlighted the importance of building connections with key figures throughout their medical education to boost students’ motivation. The promotional situational awareness domain assessed students’ views on staying informed about their academic progress and understanding their educational standing. The regulation of situational interest domain explored students’ engagement in activities like gamification, linking subjects to personal interests, and incorporating enjoyable tasks to enhance their attraction and interest in learning. The self-consequencing domain investigated students’ approaches to setting personal commitments to complete tasks or meet academic responsibilities, along with the use of rewards or penalties. Finally, the preventional situational awareness domain examined students’ perspectives on actions such as learning from negative role models or seeking information about their future educational opportunities.

We conducted a pilot testing on 30 participants, removed from the final analysis, to examine the internal validity of the MSMQ tool. The Cronbach α for all combined subscales was 0.94. The reliability indices for the regulation of value, environmental structuring, regulation of relatedness, promotional situational awareness, regulation of situational interest, self-consequencing, and preventional situational awareness strategies were as follows: 0.85, 0.85, 0.78, 0.81, 0.88, 0.89, and 0.73, respectively.

### Statistical analysis

All collected data were coded, and analyzed using SPSS (IBM Corp, IBM SPSS Statistics for Windows, Version 23.0, Armonk, NY, USA). Categorical variables were presented as frequencies [n (%)], while continuous variables were presented as means ± standard deviations. Group comparisons were evaluated using the t-test and ANOVA and their non-parametric counterparts of Mann-Whitney U/Kruskal-Wallis tests whenever applicable. Multiple linear regression was performed to identify predictors of meta-motivational strategy use (e.g., GPA, gender, living arrangements). Assumptions of the linear regression models were examined as follows: linearity was examined using scatter plots, equal variances was examined through observing a random allocation between residuals and predicted outcomes, Q-Q and P-P plots were utilized to assess for multivariate normality, autocorrelation was examined using the Durbin-Watson test with an optimal range between 1.8 and 2.2, and multi-collinearity was examined using the variance inflation factor at a threshold of < 2.5.

## Results

A total of 409 medical students were included in the final analysis. The mean age for the included sample was 20.9 ± 2.0 years. Male-to-female ratio was 1.14-to-1.0 (53.3% to 46.7%). The majority of participants resided with either family or friends (66.0%), had no chronic diseases (88.5%), and were first-generation medical students (70.4%). When asked about their influence on their medical career, 83.6% responded that it was their own choice, while 16.4% reported joining due to familial consensus.

About 64.5% of participants are in their first 3 years of undergraduate studies. Most participants had a GPA of “good” or above (95.6%) and have had never failed a university module (61.6%). When asked about research publications, only 18.4% of participants had at least one publication. Only 1.5% of the included sample had over 5 publications as of time responding to this study. Characteristics of included participants are presented in Table 1.

**Table 1:** Characteristics of included participants

Variable	Category	*N*	%
Gender	Female	191	46.7%
	Male	218	53.3%
Year of study	1st	68	16.6%
	2nd	100	24.4%
	3rd	96	23.5%
	4th	51	12.5%
	5th	68	16.6%
	6th	20	4.9%
	7th	6	1.5%
GPA	Below good (< 3.0)	18	4.4%
	Good (3.0	140	34.2%
	Very good (3.0 < x < 3.6)	168	41.1%
	Excellent (> 3.6)	83	20.3%
Status	First-Generation	121	29.6%
	Not First-Generation	288	70.4%
Choice to join medicine	Family’s choice	67	16.4%
	My choice	342	83.6%
Number of publications	0	333	81.4%
	1–3	67	16.4%
	4–5	3	0.7%
	5+	6	1.5%
Number of failed modules	0	252	61.6%
	1–3	109	26.7%
	4–5	39	9.5%
	5+	9	2.2%

### Meta-motivational strategies in medical students questionnaire

**Fig. 1:**
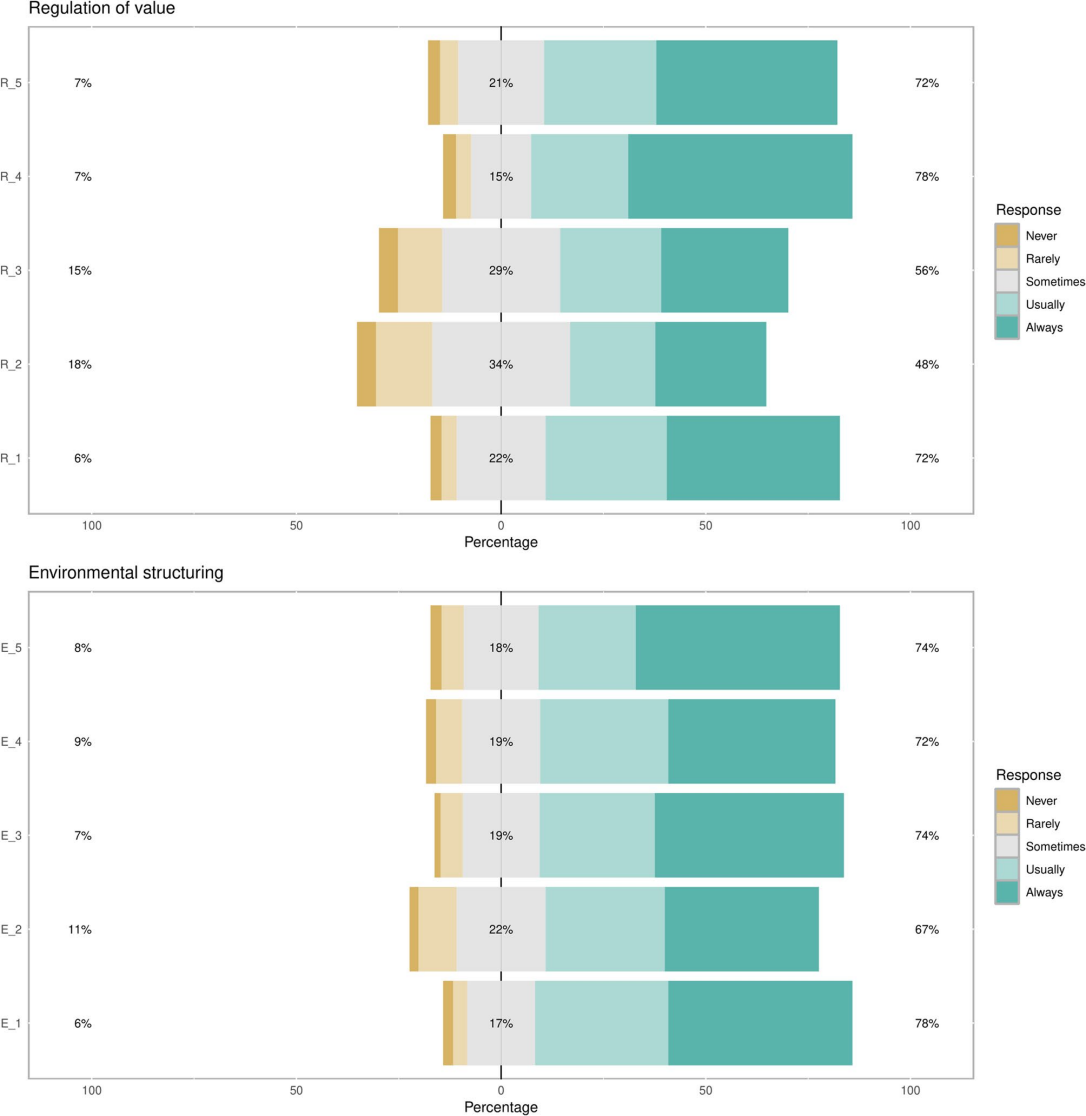
Responses to regulation of value and environmental structuring domains

In terms of regulation of value strategy, the majority of students consistently think about the value and importance of their educational materials for future purposes (72%), remind themselves of such value (78%), and relate their materials to educational profession to sustain their perception of their importance (72%). In terms of environmental structuring, the majority of students had favorable responses to all items as more than 67% would consistently attempt to create a favorable studying environment, eliminate external distracting factors, and create a state of mental readiness within their environment. Figure 1 demonstrates participants’ responses to the ‘regulation of value’ and ‘environmental structure’ domains.

**Fig. 2:**
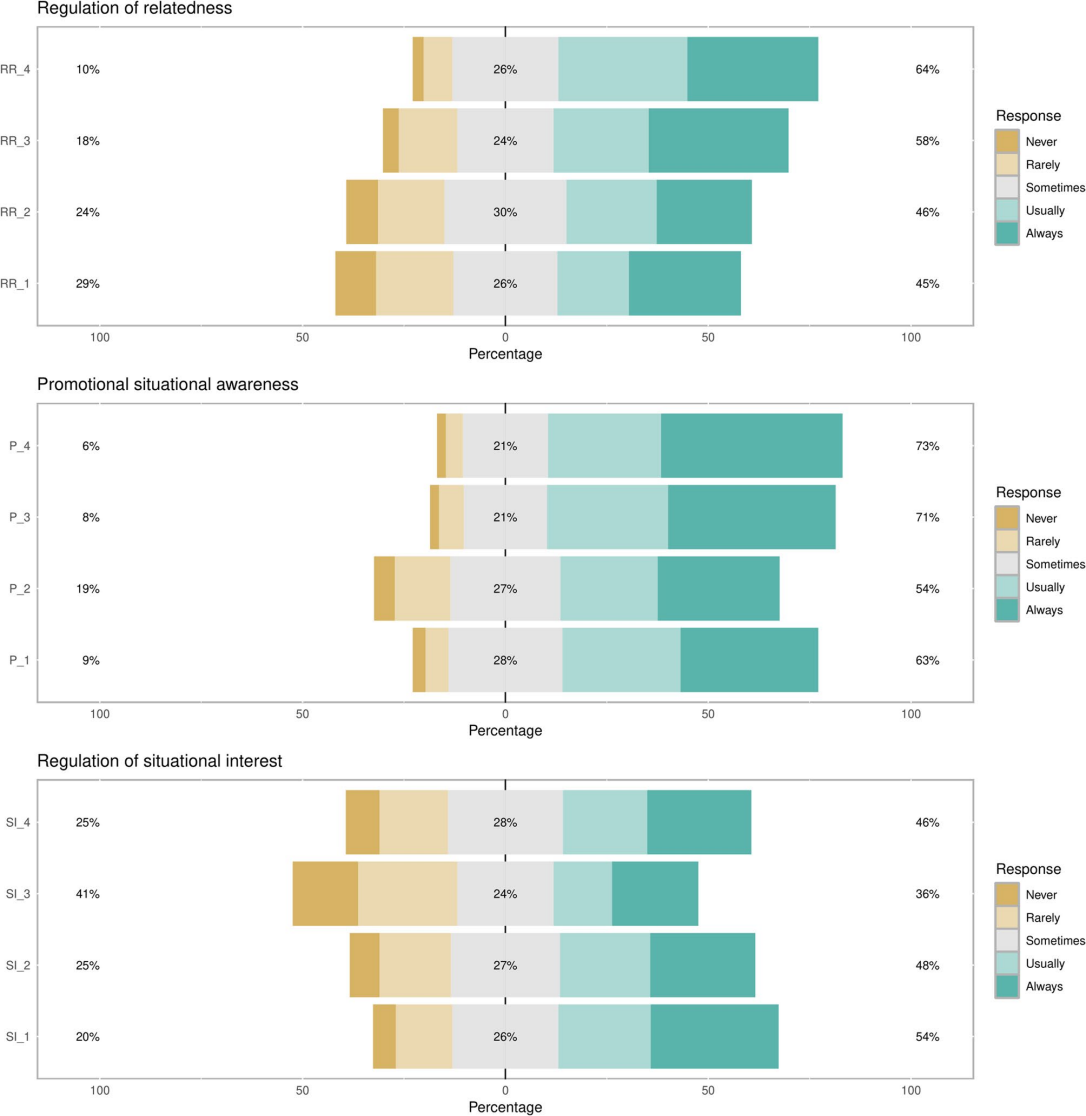
Responses to regulation of relatedness, promotional situational awareness, and situational interest domains

With respect to regulation of relatedness strategies, 64% of students emphasize with peers, family, or patients about educational materials, while 58% consistently ask for advice from senior peers. Consultations with professors were the least consistently utilized strategy as 29% had rarely or never attempted such course of action. In terms of promotional situational awareness, most students consistently introspect about their performance (73%) and their strengths and weaknesses (71%). On the other hand, only 51% would identify professors or senior students as academic role models. Finally, there was no dominant strategies in terms of regulation of situational interest. Figure 2 demonstrates participants’ responses to the ‘regulation of relatedness’, ‘promotional situational awareness’, and ‘regulation of situational interest’ domains.

**Fig. 3:**
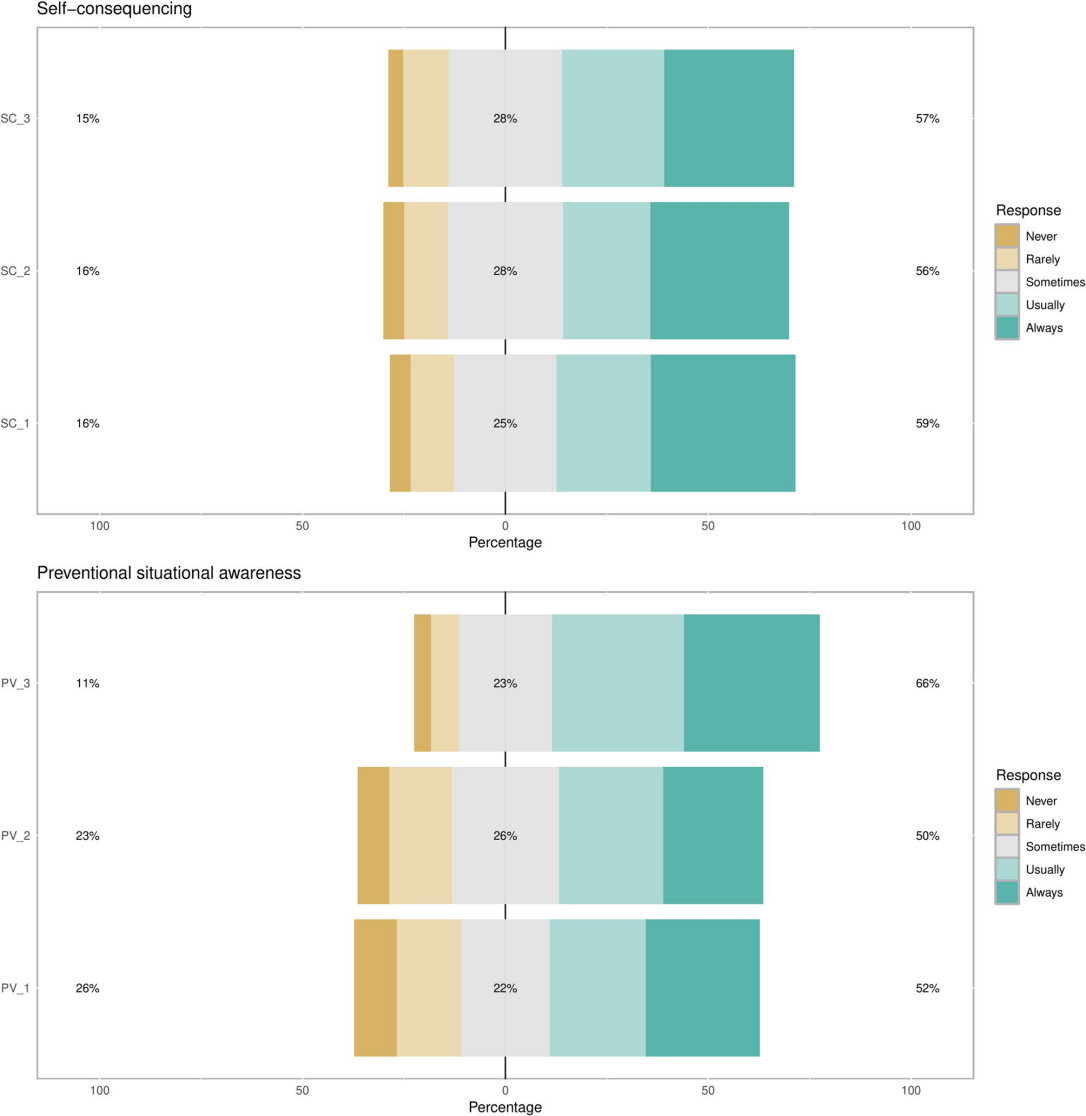
Responses to self-consequencing and preventional situational awareness regulation domains

Over 55% of included participants consistently engaged in self-consequencing strategies through positive reinforcement. Finally, with regards to preventional situation awareness strategies, over 50% of participants consistently identify bad professors or unsuccessful students for reverse role modeling, attempt to mirror the knowledge, style, and characteristics of relevant professors, and be aware of the accepted norms for a set educational situation. Figure 3 demonstrates participants’ responses to the ‘self-consequencing’ and ‘preventional situational awareness’ domains.

### Bivariate analysis

In terms of differences between strategies scores, male medical students had significantly higher regulation of relatedness scores compared to their female counterparts (MD: −0.79; 95%CI: −1.51 – −0.06; *p* = 0.019). Furthermore, participants who live alone demonstrated significantly higher environmental structuring (MD: 1.16; 95%CI: 0.34–1.99; *p* = 0.001) and self-consequencing scores (MD: 0.68; 95%CI: 0.28–1.33; *p* = 0.024) compared to their counterparts living with family or friends.

**Fig. 4:**
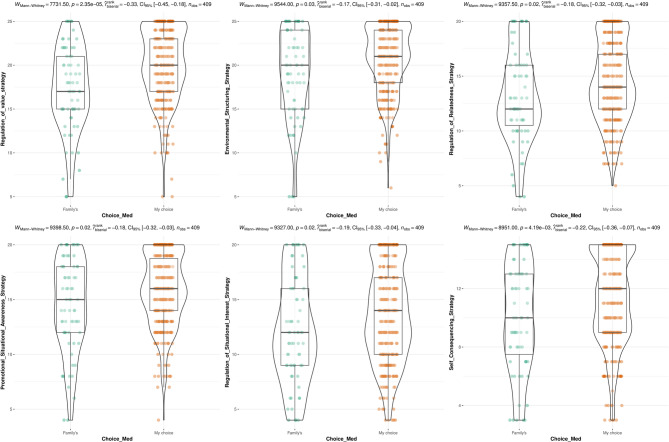
Violin plot of differences in regulation strategies in students and whether they enrolled in medicine on their own or their family’s choice

**Fig. 5:**
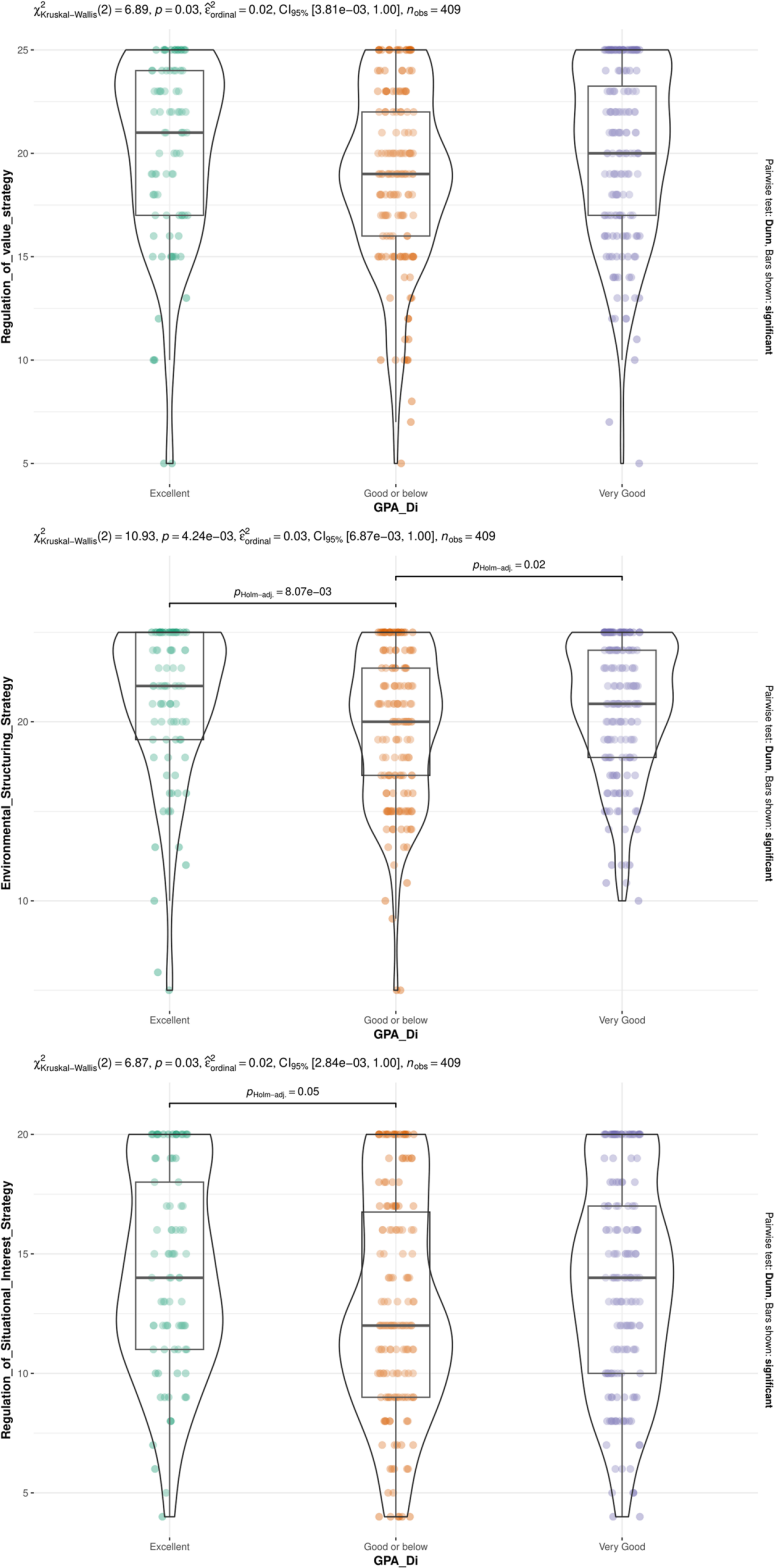
Differences of meta-motivational domains across academic performance (GPA)

While there were no significant differences in any strategy between first-gen medical students and their counterparts, participants who entered medicine out of their own volition had significantly higher scores across all meta-motivational strategies for the exception of preventional situational awareness (Fig. 4). Furthermore, published students exhibited significantly higher regulation of value (MD: −0.98; 95%CI: −2.05–0.09; *p* = 0.039), regulation of relatedness (MD: −0.97; 95%CI: −1.90 – −0.03; *p* = 0.029), and regulation of situational interest (MD: −1.25; 95%CI: −2.34 – −0.16; *p* = 0.021) scores compared to their non-published counterparts. Finally, there were significant differences in regulation of value, environmental structuring, and regulation of situational interest scores across GPA strata (Fig. 5).

## Discussion

In our study, we highlighted the meta-motivational strategies employed by medical students in Jordan across multiple domains. We explored distinct patterns in self-regulation approaches to academic motivation in relation to performance and many sociodemographic factors, underscoring the complexity of such phenomena and highlighting areas for potential intervention to further improve student well-being and performance.

The seven strategies identified in our study mirror frameworks from published international studies [15, 17]. Situational interest strategies were significantly less utilized by participating students. Situational interest is the spontaneous and context-dependent attraction to specific learning tasks and is heavily influenced by the delivery and design of educational content. Traditional lecture-based environments are the most prevalent among Jordanian medical schools [19]. In such settings, there is often limited utilization of interactive teaching methods that provoke immediate interest. Such a lack of engagement results in diminished situational interest among students [20, 21]. This reliance on traditional, fast-paced standardization of medical education could be attributed to the previous changes in admission policies across Jordanian medical schools. These changes promoted significantly higher admission numbers without improving nor expanding the infrastructure of medical schools. Such a policy, which is currently being reverted, may have forced faculty members to utilize teaching tools that may lack individuality or engaging tasks.

Educators should strive to creatively alter their methods to match the needs of modern students. Students in the social media age are already significantly imprinted by the fast-paced, addictive designs of interactive media at both the academic and emotional levels [22, 23]. Gamification of medical education, while not fully validated and standardized, have been recognized in enhancing collaboration, promoting student engagement, sustaining students’ attention and retention, improving analytic and clinical reasoning skills, as well as, familiarizing them with practice through emulation [24, 25]. Serious games, escape rooms, simulators, gamified digital platforms, and AI-interactive are some of the most widely used gamification techniques in medical education [25]. Nonetheless, concerned parties should take into consideration design under resource scarcity, the underutilization of cognitive theories in present evidence, and limited longitudinal investigations.

There is a stark dominance of value regulation and environmental structuring among our participants, as these were the most commonly reported meta-motivational strategies. Value regulation involves the active role a student takes to enhance the perceived importance of their academic task to align them with personal goals and future professional aspirations [15, 26]. This approach has been shown to significantly predict self-efficacy, self-relevant value, and intrinsic motivation, which are essential elements for long-term academic success and engagement [17]. Students, in turn, may instinctively turn to these techniques to stay motivated, especially in a high-stakes environment, such as that of medical school. Furthermore, in Jordanian societies, meeting familial and societal expectations in those regards is crucial as well as values are often shaped by cultural norms, such as the prioritization of health and education [27].

On the other hand, environmental structuring pertains to the deliberate action of organizing one’s physical and social study environment to reduce distractions and enhance focus. The high emphasis on environmental structuring reflects the adaptive response that demanding fields like medicine often require, as students develop structured approaches to manage the workload. Students who live alone, for example, scored higher in utilizing this strategy, likely due to the need for self-regulation in independent settings compared to their counterparts [28].

Importantly, students who enrolled in medicine out of their own choice had scored significantly higher on all meta-motivational domains with the exception of preventional situational awareness. The Self-Determination Theory posits that intrinsic motivation fosters proactive engagement with specified learning goals. Students who self-selected to enroll in medicine likely perceive greater personal relevance in their studies compared to their counterparts [29]. Conversely, preventional situational awareness involves behaviors to avoid negative outcomes. Students who exhibit such behaviors may be more likely to be motivated by external pressures. The published literature echoes our findings as aligning tasks and educational goals with personal values is recognized to enhance meta-motivational regulation and is the key to successful life-long learning in medicine [6, 17].

Students who actively engaged in research opportunities have scored significantly higher in regulation of relatedness, value, and situational interest. Engaging in research often fosters working in teams with individuals in different stages of their careers. Such environments require cooperation between different medical students which may explain tendencies of those students to engage in regulation of relatedness [30]. Furthermore, successfully navigating the research process fosters autonomy, competence, and relatedness which are key pillars of sustaining intrinsic motivation. In addition, students often engage in research topics that align with their personal interests or clinical curiosity. Students who find meaning in their research topics are more likely to sustain effort despite challenges, as their work resonates with their identity or aspirations, especially when these efforts translate into a peer-reviewed publication [18, 26, 31].

Interestingly, while completing a full-fledged manuscript is a mandatory requirement across all Jordanian medical schools and research offices were or are already being established, conducting research that is meaningful to students’ educational outcomes is met with a plethora of barriers at both the undergraduate and post-graduate levels [31, 32]. The positive attitudes of students towards research are mostly due to the intention to migrate post-completing their medical training and the need to enroll in overseas competitive programs [33]. However, the current curricula are strictly traditional, heavily relying on lectures throughout the student day without any form of engaging activities [34]. This is further compounded by the lack of protected time, mentorship, or proper research training.

Interestingly, our results show that there is no statistical difference between first-generation medical students and legacy medical students. First-generation students may develop universal adaptive strategies to overcome systemic barriers they often face. Despite this disproportionate adversity, they might adopt similar meta-motivational strategies compared to their non-first-generation peers to navigate the demands of medical school. Furthermore, almost all of the participants chose medicine autonomously, which significantly correlated with higher overall strategy scores. First-generation students, despite facing potential socioeconomic challenges, may still exhibit strong intrinsic motivation if their career choice aligns with their personal aspirations, further reducing differences in adopting meta-motivational strategies [15, 35].

However, the lack of differences between first-generation and continuing-generation medical students is not surprising. Aboudeif et al. observed that while the initial academic performance of first-generation students might be lower compared to their counterparts, they develop enough resilience enabling them to catch up and even surpass those from families well established in medicine [36]. Zhang et al. notes that self-regulated learning is shaped by personal, social, and contextual factors [37]. Within the Jordanian context, traditional learning provides the same quality and degree of education to all. Moreover, contextual supports (e.g., mentorship) are already uniformly sparse. In other words, the “playing field” is level for all sects of students. Additionally, the societal pressures and fairly linear educational outcomes (i.e., higher grades) are similar across all enrolled medical students. These factors inherently eliminate the presence of privileged routes and influence meta-motivational strategies in a similar fashion.

We note gender differences in utilizing regulation of relatedness score, with males having significantly higher scores. Traditional gender roles in conservative societies may encourage males to engage more actively in peer collaboration and mentorship-seeking behaviors compared to females [38]. Male students might align their intrinsic motivation with collaborative learning strategies. They may also prioritize networking or peer discussions as a means to reinforce their understanding, while females might focus more on independent study. This aligns with findings that males often report higher intrinsic motivation but paradoxically lower academic performance, suggesting a reliance on social learning to compensate [39]. Certain traits, namely confidence, may also be at play. Studies indicate that female medical students often report lower confidence and higher self-doubt compared to males, even when academic performance is comparable. This could lead to reduced reliance on regulation of relatedness strategies as females may hesitate to admit uncertainty or ask for help [40, 41].

However, the elephant in the room is Jordan’s conservative, dominantly Muslim, culture, by which gender norms and traditions shape interactions. Female students are less likely to engage in both educational and clinical activities as females are often expected to avoid mixed-gender contact [42]. Within academic settings, gender is a source of bias, as female students consistently reported fewer same-sex mentors and role models within their undergraduate medical education [31]. In short, these factors present female students with less opportunities to utilize relatedness strategies, shifting their motivation into more individualistic methods.

### Limitations

The study’s cross-sectional nature precludes establishing causal relationships between meta-motivational strategies and outcomes. As the study is designed to capture data at a single point in time, our findings may not capture how such strategies fluctuate over different academic phases or examination periods. Longitudinal research is needed to assess how these strategies evolve over time or influence long-term success. Furthermore, reliance on self-reported questionnaires introduces potential recall bias and social desirability bias. Although we surveyed six major universities, we did not account for differences in teaching methods, curricular design, or assessment practices across these institutions, which may influence both the availability of and students’ propensity to utilize certain strategies. Moreover, we did not include qualitative data to further contextualize how students may interpret and employ each meta-motivational strategy, which may affect how nuanced factors, such as specific classroom dynamics or individual stressors, are at play in this relationship. Finally, our sampling methods were non-random. Complemented by a cross-sectional design, these methods may result in the under/over-representation of certain sects of medical students, overlooking important subgroups. Moreover, due to the dissemination of the survey instrument using social media platforms, selection bias could’ve occurred as more motivated students were more likely to complete the form and potentially lead to a skewed understanding of students’ meta-motivational tendencies. Future research should focus on utilizing mixed-method designs and have assessments at multiple timepoints to address these limitations to be more comprehensive in understanding how students regulate their meta-motivational strategies.

## Conclusion

This study provides novel insights into the meta-motivational strategies employed by Jordanian medical students, emphasizing their adaptive responses to academic challenges within a culturally specific context. We noted a dominance of regulation of value and environmental structuring strategies. Moreover, differences across gender and publication status were also observed.

The underutilization of situational interest strategies suggests opportunities for curricular innovation, such as integrating gamification or interdisciplinary collaboration to enhance engagement. Future researchers are encouraged to longitudinally examine the temporal changes in meta-motivational strategies, their association with academic performance, and deconstruct motivational components within commonly utilized strategies. Moreover, mixed-method endeavors are required to identify the phenomenological experiences across motivational components and their relationship to students’ academic interest. Finally, concerned policy makers could hold training courses for faculty members, advisors, and students, which could help enhance the effectiveness of current strategies while also promoting self-awareness of its underutilized counterparts.

## Data Availability

All data/datasets associated with this project will be provided at a reasonable request from the corresponding author.

## Abbreviations

SDT: Self-determination theory
MSMQ: Meta-motivational Strategies in Medical Students Questionnaire
